# eccCL: parallelized GPU implementation of Ensemble Classifier Chains

**DOI:** 10.1186/s12859-017-1783-9

**Published:** 2017-08-17

**Authors:** Mona Riemenschneider, Alexander Herbst, Ari Rasch, Sergei Gorlatch, Dominik Heider

**Affiliations:** 1 Department of Bioinformatics, Straubing Center of Science, Petersgasse 18, Straubing, 94315 Germany; 2 Institute of Computer Science, University of Münster, Einsteinstr. 62, Münster, 48149 Germany; 3 Wissenschaftszentrum Weihenstephan, Technische Universität München, Alte Akademie 8, Freising, 85354 Germany; 4 Present Address: Department of Mathematics and Computer Science, University of Marburg, Hans-Meerwein-Str. 6, Marburg, 35032 Germany

**Keywords:** Classifier chains, Multi label classification, High performance computing

## Abstract

**Background:**

Multi-label classification has recently gained great attention in diverse fields of research, e.g., in biomedical application such as protein function prediction or drug resistance testing in HIV. In this context, the concept of Classifier Chains has been shown to improve prediction accuracy, especially when applied as Ensemble Classifier Chains. However, these techniques lack computational efficiency when applied on large amounts of data, e.g., derived from next-generation sequencing experiments. By adapting algorithms for the use of graphics processing units, computational efficiency can be greatly improved due to parallelization of computations.

**Results:**

Here, we provide a parallelized and optimized graphics processing unit implementation (eccCL) of Classifier Chains and Ensemble Classifier Chains. Additionally to the OpenCL implementation, we provide an R-Package with an easy to use R-interface for parallelized graphics processing unit usage.

**Conclusion:**

eccCL is a handy implementation of Classifier Chains on GPUs, which is able to process up to over 25,000 instances per second, and thus can be used efficiently in high-throughput experiments. The software is available at http://www.heiderlab.de.

## Background

Multi-label classification (MLC) has gained significant attention in recent years in diverse fields of research, e.g., in protein function prediction [1] and text categorization [2], as well as in biomedical research [3–5]. For instance, in recent work the MLC concept of classifier chaining was applied to the problem of drug resistance prediction in HIV [6].

**Fig. 1:**
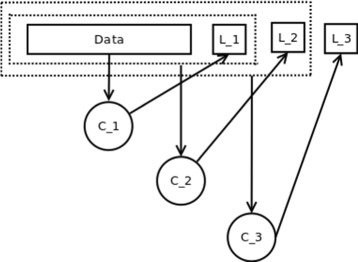
General concept of classifier chanining. In general, classifier *C*_*i*_ knows the labels *L*_0_,...,*L*_*i*−1_ of classifiers *C*_0_,...,*C*_*i*−1_ in training process and in classification process the results of classifiers *C*_0_,...,*C*_*i*−1_. Here, the concept of classifier chaining is depicted for three class labels

The concept of Classifier Chains (CC) is a generalization of binary classification. In MLC each instance is associated with a set of labels instead of one single label as in binary classification. Formally, let *L*={*l*_1_,…,*l*_*m*_} be a set of class labels and *Y* the power set of labels defining the possible label combinations of *L*. Let *X* be the input space, where each vector *x* represents an instance, e.g., a protein sequence, which is associated with labels of *Y*. The idea of CC is to generate a single classifier for each *l*∈*L* and to link the single classifiers along a chain. The general concept of classifier chaining is exemplarily shown for three labels in Fig. 1. One major advantage in classifier chaining is that interdependencies between class labels can be modeled, e.g., in the case of drug resistance prediction, where resistance to one drug type might also be indicative of resistance against another drug. However, the order in CC may have an influence on the accuracy of prediction due to error propagation [7]. An extension to overcome these effects are Ensemble Classifier Chains (ECC) [8]. In this approach *k* classifier chains are trained with each chain in random order and with different subsets of training data. The prediction outcome is then combined by a voting scheme, e.g., by thresholding the prediction of each label and chain. Overall, the concept of classifier chaining has been shown to improve prediction accuracy, particularly when applied as ECC [9, 10].

However, today it is necessary to process large amounts of data which typically comes with big data problems, e.g., in biomedical research the usage of data generated by next-generation sequencing technologies or functional magnetic resonance imaging [11, 12] is still challenging as current available implementations lack computational efficiency. Therefore, parallelized architectures, especially graphics processing unit (GPU) implementations might provide remedy in regards of expensive computing time [13, 14]. For example, Olejnik et al. [15] recently published a GPU implementation to predict the co-receptor usage in HIV. Whereas the CPU implementation [16] was able to classify only few instances per second, the parallelized and optimized GPU version processes a significantly increased amount of instances per second.

Here, we provide a parallelized implementation of CC and ECC optimized for parallelized GPU usage. Our implementation is able to classify over 25,000 instances per second, whereas the sequential implementation on the CPU provided by the Mulan library (http://mulan.sourceforge.net) is able to classify only 360 instances per second.

## Implementation

Our software is implemented in Java using the Lightweight Java Game Library (LWJGL) (http://www.lwjgl.org) enabling the development of parallel computing applications based on OpenCL. The software can be used in Java as library or CLI-application or with R (http://www.r-project.org) by installing the R package eccCL. For the communication between R and Java the rJava package is used. As a base classifier, we implemented random forests for GPU usage.

A random forest [17] is an ensemble learning method for classification and regression. A random forest trains several decision trees on a subset of the original dataset. Major advantages of random forests are the control of overfitting and the improved prediction accuracy which is achieved by the combination of prediction results of each individual tree to a final decision. Parallelization is achieved in two ways: First, each decision tree within a random forest is built in a concurrent task in the training phase. Second, in the classification phase each instance is classified in a concurrent task. In contrast to the Mulan library, eccCL is able to use OpenCL. This implicates that the subsets for each node in training are not dynamically created as this is not possible in OpenCL, compared to Mulan. Furthermore, each tree has the exact same number of nodes and the exact same depth, thus the classifiers can be stored in a single array and the position of each node can be calculated. Additionally, all instances are stored in a single buffer. Furthermore, instead of generating random subsets dynamically in the training phase, the index positions of the instances are stored in a separate array and reordered in a randomized manner for each node, due to the fact that all arrays in OpenCL need to have a fixed size at compile time.

## Results and discussion

We developed a GPU framework for modeling CC and ECC. The software was evaluated on an Intel Xeon E5-1620 with 4 cores and an NVIDIA Tesla K20c with 2496 streaming processors. The data sets for the evaluation of our implementation were taken from different research areas. The *NNRTI* and *PI* dataset are from the realm of drug resistance prediction [18] in HIV. The data sets *emotions* [19], *scene* [20], and *yeast* [21] are received from the Mulan project (http://mulan.sourceforge.net) which provides an implementation for the usage of CC and ECC, however, implemented in a non-parallelized manner.

The software can be used via Java on command line with parameter settings or in R by installing the R package eccCL. The software can be downloaded at the authors homepage (http://www.heiderlab.de). After downloading, the R package can be installed using the R command within the R command line: install.packages(‘/path/to/package/eccCL.tar.gz’, repos=‘NULL’). In the following we demonstrate how to build an ECC with an ensemble size of 20 chains and a forest size of 64 within R:library(eccCL)

# Load file (.arff and.xml format

must be available)

data <- eccCLloadWekaFile

(‘home/temp/example’)

# Build classifier

ecc <- eccCLbuildFromObject(data,

ensembleSize=20, forestSize=64)

# Classify data

out <- eccCLclassifyObject(ecc, data)

# Get classification results

res <- eccCLgetResults(out)

# Save and load classifier

eccCLstore(ecc,

‘/home/temp/classifier.stored’) ecc

<- eccCLload(‘/home/temp/classifier.

stored’)

The data format should be in.arff and.xml format according to the Mulan library. The files must be available in the given path. In the building process of the classifier, the ensemble size and forest size can be set individually. The classifier can be saved and loaded again for later classification tasks. Equivally, the following line represents the usage with Java as a shell command using the jar-file:

java -jar EccCL.jar -inpData /path/to/dataset/NNRTI -eccES 20 -eccFS 64 -evalAllLabels

The classifier will be trained and a classification will be performed. A classification task without a training process on a trained and saved classifier can be executed with the command:

java -jar EccCL.jar -inpData /path/to/dataset/NNRTI -classOnly /path/to/trainedClassifier

Table 1 provides a speed-up comparison between our GPU implementation and the Mulan framework with the usage of 20 ECC and 64 trees per random forest. Additionally, Table 2 demonstrates the number of instances classified per second with eccCL compared to the Mulan framework with respect to an increasing number of instances. Overall, our GPU implementation shows a speed-up of an order of magnitude in computation times. The prediction accuracy shows no difference between the GPU implementation and the models of the Mulan framework, however, slightly dependent on the parameter settings.

**Table 1:** Comparison between our GPU implementation and the non-parallelized Mulan framework for the classification of instances based on different data sets with different counts of instances and labels

	#Instances	Mulan	GPU	Speed-up
NNRTI	715	1563.7	109	14x
PI	662	1998.6	128.2	15x
Emotions	593	1577.3	157.7	10x
Scene	2407	8920.3	300.9	29x
Yeast	2417	270736.2	379.2	71x

The runtimes are shown in milliseconds

**Table 2:** Instances classified per second with increasing number of bootstrapped instances exemplarily shown for the PI dataset

#Instances	Mulan	GPU
1000	357	2,516
10,000	342	11,510
100,000	352	25,851
1,000,000	362	26,266

Our software can be used on standard desktop PCs and with OpenCL-ready graphics cards, whereas in general currently available GPUs of almost all manufacturers support OpenCL. eccCL needs Java (version 8.0) and OpenCL (version 1.2) installed. Furthermore, R (version 3.0) and the rJava package (version 3.2) have to be installed in advance for the usage of eccCL with R interface. Dependent on the platform, the OpenCL implementation can be used and in case OpenCL is not installed a parallelized Java implementation can be executed, however, on the CPU. eccCL runs on Linux and Mac OS. Overall, the software is easy to handle and no special hardware, i.e., a cluster or high-end server is needed. Currently, the eccCL package provides the random forest classifier in a parallelized manner. Random forests can be used as a classifier chain classifier and as an ensemble classifier chain classifier. In the future, we will work on further classifier implementations and will make them available within our package.

## Conclusion

We provide an R-package and a Java version of a parallelized and optimized GPU implementation of Classifier Chains and Ensemble Classifier Chains. The software is able to classify up to over 25,000 instances per second and thus can efficiently speed up the classification process in high-throughput experiments.

## Availability and requirements

**Project name:** eccCL**Project home page:** http://heiderlab.de **Operating system(s):** Linux, Mac OS**Programming language:** Java (≥ 8.0), R (≥ 3.0), (optional) OpenCL (≥ 1.2)**License:** GPL (≥ 2)**Any restrictions to use by non-academics:** none

## Abbreviations

CC: Classifier chains
CLI-application: Command-line interface application
CPU: Central processing unit
ECC: Ensemble classifier chains
GPU: Graphics processing unit
HIV: Human immunodeficiency virus
MLC: Multi-label classification
NNRTI: Non-nucleoside reverse transcriptase inhibitor
PI: Protease inhibitor
